# A comparison of FreeStyle Libre 2 to self-monitoring of blood glucose in children with type 1 diabetes and sub-optimal glycaemic control: a 12-week randomised controlled trial protocol

**DOI:** 10.1007/s40200-021-00907-y

**Published:** 2021-10-05

**Authors:** Sara Styles, Ben Wheeler, Alisa Boucsein, Hamish Crocket, Michel de Lange, Dana Signal, Esko Wiltshire, Vicki Cunningham, Anita Lala, Wayne Cutfield, Martin de Bock, Anna Serlachius, Craig Jefferies

**Affiliations:** 1 Department of Human Nutrition, University of Otago, Dunedin, New Zealand; 2 Department of Women’s and Children’s Health, University of Otago, Dunedin, New Zealand; 3 Paediatrics, Southern District Health Board, Dunedin, New Zealand; 4 Health, Sport and Human Performance, School of Health, University of Waikato, Hamilton, New Zealand; 5 Centre for Biostatistics, Te Pokapū Tatauranga Koiora, Division of Health Sciences, Dunedin, New Zealand; 6 Paediatric Diabetes and Endocrinology, Starship Children’s Health, Auckland, New Zealand; 7 Liggins Institute, The University of Auckland, Auckland, New Zealand; 8 Department of Paediatrics and Child Health, University of Otago, Wellington, Wellington, New Zealand; 9 Capital & Coast District Health Board, Wellington, New Zealand; 10 Northland District Health Board, Whangarei, New Zealand; 11 Paediatrics, Bay of Plenty District Health Board, Tauranga, New Zealand; 12 Department of Paediatrics, University of Otago, Christchurch, New Zealand; 13 Canterbury District Health Board, Christchurch, New Zealand; 14 Psychological Medicine, The University of Auckland, Auckland, New Zealand; 15 Department of Paediatrics and Child Health, University of Otago, Wellington, New Zealand

**Keywords:** Children, Intermittently scanned continuous glucose monitoring, Glycaemic control, Type 1 diabetes, FreeStyle Libre 2, Self-monitoring of blood glucose

## Abstract

**Purpose:**

Frequent glucose monitoring is necessary for optimal glycaemic control. Second-generation intermittently scanned glucose monitoring (isCGM) systems inform users of out-of-target glucose levels and may reduce monitoring burden. We aim to compare FreeStyle Libre 2 (Abbott Diabetes Care, Witney, U.K.) to self-monitoring of blood glucose in children with type 1 diabetes and sub-optimal glycaemic control.

**Methods:**

This open-label randomised controlled trial will enrol 100 children (4–13 years inclusive, diagnosis of type 1 diabetes ≥ 6 months, HbA1c 58–110 mmol/mol [7.5–12.2%]), from 5 New Zealand diabetes centres. Following 2 weeks of blinded sensor wear, children will be randomised 1:1 to control or intervention arms. The intervention (duration 12 weeks) includes second-generation isCGM (FreeStyle Libre 2) and education on using interstitial glucose data to manage diabetes. The control group will continue self-monitoring blood glucose. The primary outcome is the difference in glycaemic control (measured as HbA1c) between groups at 12 weeks. Pre-specified secondary outcomes include change in glucose monitoring frequency, glycaemic control metrics and psychosocial outcomes at 12 weeks as well as isCGM acceptability.

**Discussion:**

This research will investigate the effectiveness of the second-generation isCGM to promote recommended glycaemic control. The results of this trial may have important implications for including this new technology in the management of children with type 1 diabetes.

**Trial registration:**

This trial was prospectively registered with the Australian New Zealand Clinical Trials Registry on 19 February 2020 (ACTRN12620000190909p) and the World Health Organization International Clinical Trials Registry Platform (Universal Trial Number U1111-1237-0090).

## Background

In New Zealand, there are an estimated 2,500 youth aged 0–18 years living with type 1 diabetes [1–3]. New Zealand has one of the highest rates of paediatric diabetes in the world, with the incidence growing annually [4]. Internationally, only one in four children with diabetes achieve international standards of glycaemic control (HbA1c < 58 mmol/mol [< 7.5%]) [5–7]. This increases their risk for short and long-term diabetes complications as shown by the Diabetes Care and Control Trial [8–10].

Frequent and timely self-monitoring of blood glucose (SMBG) is essential for guiding diabetes management decisions and keeping glucose levels in a safe range. Conventional SMBG involves finger-stick blood tests six or more times each day [11]. Children may infrequently perform SMBG because of social pressure to not be seen as ‘different’ [12], physical discomfort from pricking their fingers, and the technology is not user friendly (requires multiple steps to obtain a reading) [13].

Real-time continuous glucose monitoring (rtCGM) and intermittently scanned CGM have significant advantages over SMBG [14]. rtCGM systems use a subcutaneous glucose sensor to transmit and display a continuous stream of real-time interstitial glucose data to a pump/reader. Despite rtCGM systems being an accurate and effective glucose monitoring tool, like other diabetes technologies they are costly which can limit, or lead to inequity in uptake, and alarms can contribute to alarm fatigue and subsequent discontinuation of rtCGM use [15–17]. An alternative to rtCGM is intermittently scanned continuous glucose monitoring (isCGM) technology. isCGM involves applying a small factory-calibrated sensor to the back of the upper arm to detect interstitial glucose levels and then scanning the sensor with a reader to immediately display the glucose level. As with newer versions of rtCGM, isCGM technology provides accurate glucose information for up to 2 weeks [18]. Randomised controlled studies and real-world data based on first-generation isCGM use have found evidence of better glycaemic control with use over a sustained period of time [19, 20].

First-generation isCGM is highly acceptable to children and young people with diabetes and their caregivers [21, 22]. The second-generation isCGM system (FreeStyle Libre 2) is more accurate than the previous generation and additionally provides personalisable hypoglycaemia and hyperglycaemia alarms [23]. First-generation isCGM has been associated with improved quality of life and improved glycaemic control over 3 months in children ages 5–18 years [24]. The optional alarm feature in the second-generation system may particularly benefit families of children with above recommended HbA1c given the alarms prompt action to treat above target glucose levels and provide peace of mind that below target glucose levels will be detected. There is one randomised controlled trial currently being conducted in adult patients with type 1 diabetes in the UK [25]. However, there are no randomised controlled trials of second-generation isCGM in paediatric patient populations. In adolescents and young adults with type 1 diabetes, the first-generation of isCGM was found to increase glucose monitoring compared to SMBG, but this did not translate into significant differences in glycaemic control (as measured by HbA1c) between groups at 6 months [26]. Given the ease of being able to scan (even through clothing), the reduction in SMBG testing and both hypoglycaemia and hyperglycaemia alarms, second-generation isCGM may provide an important opportunity to help children and their families improve self-management behaviours [26].

The proposed trial aims to investigate the effectiveness of the second-generation isCGM for reducing HbA1c in children above the recommended glycaemic control target compared to SMBG.

## Methods

### Study design

**Fig. 1:**
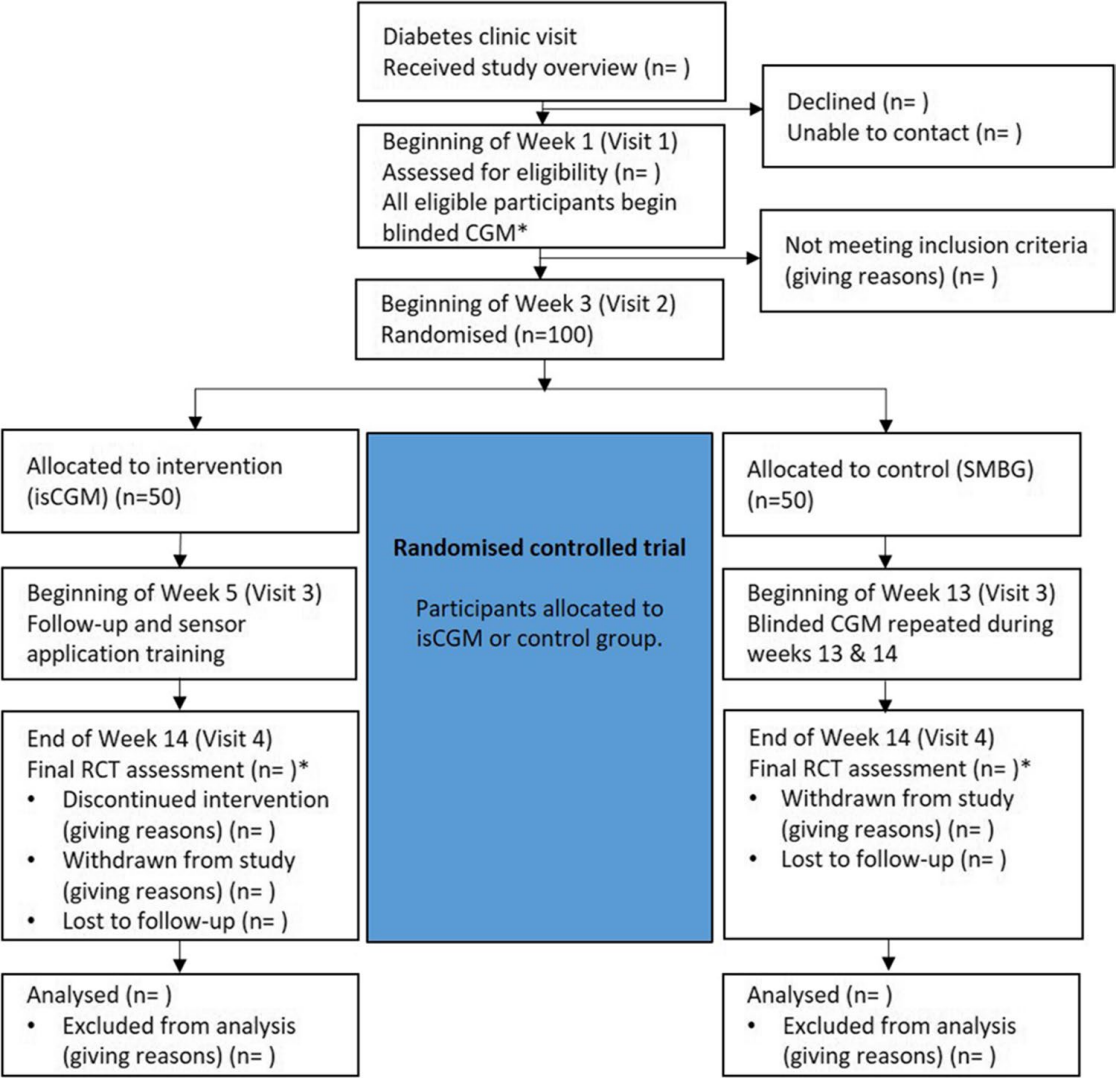
Study design. CGM, continuous glucose monitoring; isCGM, intermittently scanned continuous glucose monitoring; SMBG, self-monitoring blood glucose

This research is comprised of a multisite 12-week randomised, controlled, parallel-group trial. As shown in Fig. 1, 100 children with type 1 diabetes will be randomised to 12 weeks of standard care (control group) or standard care plus isCGM (intervention group). The study was approved by the Northern A Health and Disability Ethics Committee (ethics reference: 20/NTA/12) and Māori (indigenous New Zealanders) Research Consultation Committees in each region. Recruitment began in October 2020 and the study is expected to be completed by December 2022.

## Study procedures

### Study population and recruitment

The study will be conducted at 5 diabetes centres across New Zealand. Participants will be paediatric patients receiving standard diabetes care through district health board (DHB) diabetes services (Auckland DHB, Southern DHB, Capital Coast DHB, Bay of Plenty DHB, and Northland DHB). These diabetes services provide care for approximately 500 + children in the study age range. During routine clinical visits, eligible children will be identified by their usual paediatric endocrinologist/diabetologist/paediatrician and invited to participate. Inclusion criteria are: diagnosis of type 1 diabetes ≥ 6 months; age 4 to 13 years inclusive; on > 0.5 units of insulin/kg/day; no regular use of isCGM or CGM in the previous 3 months; and current HbA1c ≥ 58 mmol/mol and ≤ 110 mmol/mol, on day of consent. Children will not be included if they are diagnosed with any severe chronic diabetes related complications or severe medical or psychiatric co-morbidity/severe mental illness requiring ongoing treatment (e.g., diagnosed eating disorder); are participating in another study that could affect glucose measurements; or have plans to leave study site regions prior to study completion. Written informed consent will be obtained from parents/guardians, written informed assent will be obtained from participants aged 7 to 13 years, and verbal assent will be obtained from participants aged 4 to 6 years. Any participant can withdraw (or be withdrawn by their parent or guardian) from the study at any point.

### Randomisation

Prior to study commencement, a randomisation table was generated by a biostatistician using Stata 15.1 software and pre-defined parameters (pre-study HbA1c [58 to 74 mmol/mol, or ≥ 75 mmol/mol; 7.5 to 8.9%, or ≥ 9.0%], study site), and imported into the REDCap randomisation module. REDCap is a secure, web-based application designed to support data capture for research studies [27]. Participants will be randomised in a 1:1 ratio to either the control (SMBG) group or the intervention (isCGM) group at Visit 2 by research staff using the randomisation module. Participants, investigators, and study staff will not be masked to group allocation.

### Control group

All participants will continue standard diabetes care from their usual paediatric diabetes care provider. Routine diabetes clinics are attended regularly (at least every 3 months) to provide diabetes care by a multi-disciplinary team (paediatric endocrinologist/diabetologist/paediatrician, diabetes nurse specialist, dietitian, psychologist). Between scheduled study visits, participants will have the usual ability to contact their clinical team as is routine for all patients. Control group participants will continue SMBG using conventional finger stick BG testing with a glucometer and be fitted with a blinded isCGM, sensor, which they will wear for the first and final 2 weeks of the RCT.

### Intervention group

The intervention consists of a FreeStyle Libre 2 isCGM system (sensors, reader, USB cable, power adapter, user’s manual, and quick start guide) and structured education from trained research staff. Education will include sensor insertion, interpreting the readings, and optimisation of insulin dosages, if appropriate. The first sensor will be applied by research staff. Participants will insert the next sensor 14 days later under supervision (Visit 3) and for the remainder of the study. Participants will be instructed to scan a minimum of 6–10 times each day with no longer than 8 h between two scans. Research staff will set the initial recommended reader settings to 3.9 mmol/L (70 mg/dL) and 15.0 mmol/L (270 mg/dL). Research staff will access glucose data online through LibreView, a secure, cloud-based system, to generate a report of participants’ average interstitial glucose level, time above/in/below range, and scans per day at 2-, 4-, 8- and 12-weeks from isCGM commencement. If the report shows time spent 'low' is > 4% or time spent 'very low' is > 1% then the report will be forwarded to the participant's clinical team for follow-up.

As a safety precaution, participants will be instructed to perform SMBG to confirm their glucose level before therapeutic interventions or corrective action are taken if hypo- or hyperglycaemic levels (≤ 4.0 or ≥ 14.0 mmol/l) or symptoms occur.

To prevent sensor loss prior to the end of the 14-day sensor session, participants will be provided with either Rockadex (pre-cut sports tape), Hypafix® (BSN medical GmbH, Hamburg, Germany) or cohesive tape to be used to attach the sensor securely in the event the adhesive becomes loose.

## Procedures

At screening and enrolment (Visit 1, beginning of Week 1) a point of care HbA1c will be measured to confirm eligibility. Date of diabetes diagnosis for subsequent calculation of duration of diabetes (month and year will be recorded when the exact date is unknown), current insulin regimen, insulin dosing, HbA1c measurements in previous 6 months, and co-morbidities will be recorded from electronic medical records. Diabetic ketoacidosis (DKA) [28] and severe hypoglycaemia events (defined as a blood glucose value ≤ 3.9 mmol/L and resulting in loss of consciousness, a call for an ambulance and/or admission to hospital, or use of glucagon) in the past 6 months will also be recorded from electronic medical records to provide baseline estimates of frequency for these events. All participants will start blinded CGM (FreeStyle Libre Pro, Abbott) to continually measure and store glucose level data for up to 14 days [29]. This glucose monitoring system uses similar sensor technology to the FreeStyle Libre 2 system in the intervention; however, the Pro system masks all glucose data until it is downloaded at Visit 2. Participants with sensor data for at least 50% of the blinded wear period will be randomised at Visit 2. Questionnaires for the participant-reported outcomes will be administered before randomisation and at the end of the 12-week RCT.

### Outcome assessments

The primary outcome is the between group change in HbA1c at 12-weeks (i.e., end of week 14 of study). The timing of all assessments is presented in Table 1. Trained research staff will be responsible for completing assessments. Visit 2 measurements will be taken before randomisation.

**Table 1:** Outcome assessments

Assessment	Prior to randomisation	During RCT (Weeks 5, 7 & 11)	End of RCT	Ongoing
Demographics	*X*			
Anthropometry	*X*			
HbA1c	*X*		*X*	
CGM metrics	*X*	*X*	*X*	
Glucose monitoring behaviour	*X*		*X*	
isCGM acceptability			*X*	
Psychosocial assessments*	*X*		*X*	
Acute type 1 diabetes complications**				*X*

* Pediatric Quality of Life Inventory (PedsQL) 3.2 Diabetes Module, Hypoglycaemia Fear Survey (HFS), Self-Efficacy for Diabetes Self-Management (SEDM). ** Diabetic ketoacidosis, moderate and severe hypoglycaemia, issues related to glucose monitoring device use.

### Demographics

A self-administered questionnaire will collect demographic information including age, gender, ethnicity, address, and education level. Participants may choose to select more than one ethnicity; however, each person will be allocated to a single ethnic group for the purposes of statistical analyses that will be prioritised in the order of Māori, Pacific, Asian and European/Other [30]. The address where the participant lives more than 50% of the time will be used to assess their New Zealand deprivation score, which is a validated index of the relative socioeconomic deprivation of the area in which an individual lives [31].

### Anthropometry

Weight and height will be measured using standard procedures and calibrated instruments. Weight will be measured with a fixed scale (DigiTol, Toledo, Switzerland or similar) or portable scale (Tanita Corporation, Japan or similar) to the nearest 0.1 kg, with shoes and heavy clothing removed. Height will be measured to the nearest 0.1 cm, by wall-fixed stadiometer (Harpenden stadiometer, Holtain Limited, Pembs, UK or similar) or a portable stadiometer (Leicester Height Measure, Invicta Plastics Ltd., Oadby, England). Height and weight will be used to calculate body mass index (BMI)-z-scores using Centers for Disease Control and Prevention growth standards [32].

### HbA1c

Glycated haemoglobin (HbA1c) will be measured by traditionally calibrated point-of-care instrument (DCA Vantage Analyzer, Siemens Healthcare Diagnostics, Ireland) at all sites, which meets acceptance criteria of having a total CV < 3% in the clinically relevant HbA1c range [33]. In the event a value is > 130 mmol/mol (> 14%, maximum reading possible) the value will be recorded as 130.

### isCGM glucose metrics

During all follow-up visits, all retrospective glucose readings from the previous 2 weeks will be downloaded from the isCGM reader or LibreView. Hypoglycaemia (time below target) will be recorded as percentage of time below target (< 3.9 mmol/L). Time in range will be recorded as the percentage of time in the range (3.9–10.0 mmol/L) [34, 35]. Hyperglycaemia (time above target) will be recorded as percentage of time above target (> 10 mmol/L). Glucose levels < 3.9 mmol/L between 10 pm and 7am (nocturnal hypoglycaemia) will be reported to the appropriate diabetes care provider for follow-up.

### Glucose monitoring behaviour

Glucose monitoring behaviour will be defined as scanning (intervention group) or SMBG (intervention and control group), which will be determined by device downloads of glucose monitoring device data.

### isCGM acceptability

isCGM acceptability will be evaluated using a non-validated instrument adapted from previous similar research [36]. On an ordinal scale from 0 (strongly disagree) to 5 (strongly agree), participants will rate their opinion regarding the following areas: acceptability of sensor application, wear/use of the device and comparison to SMBG.

### Psychosocial assessments

Psychosocial data and overall diabetes treatment acceptance will be collected through validated self-report questionnaires completed online using (REDCap Research Electronic Data Capture) software and the order of administration will be standardised to increase reliability. Together the questionnaires will take between 30 and 45 min to complete at each time point. All questionnaires will be administered in English. Clinical care teams will be notified if participants report physical or mental health problems necessitating follow-up.

The 33-item Pediatric Quality of Life Inventory (PedsQL) 3.2 Diabetes Module is a measure of diabetes-specific health-related quality of life that assesses participant’s and parent’s/guardian’s perceptions of the participant’s diabetes-specific symptoms and management challenges during the past month [37]. The PedsQL 3.2 Diabetes Module measures five domains: Diabetes Symptoms, Treatment Barriers, Treatment Adherence, Worry and Communication. Participant self-report forms are specific for ages 5–7, (young child), 8–12 (child), and 13–14 (adolescent). The parent proxy form is specific to ages 2–4 (toddler), 5–7 (young child), 8–12 (child), 13–14 (adolescent). The PedsQL 3.2 Diabetes Module Diabetes Symptoms and Diabetes Management Summary scores have demonstrated excellent measurement properties and are recommended as useful standardised patient-reported outcomes of diabetes symptoms and diabetes management in clinical research in children with type 1 diabetes [37]. Items are rated from 0 (never a problem) to 4 (almost always a problem). Item ratings are then reverse scored and linearly transformed to a 0–100 scale, with higher scores reflecting a better quality of life.

The Hypoglycaemia Fear Survey for Children (HFSC) is a 25-item instrument adapted from the adult HFS [38]. The HFSC will be completed by children aged 6 years and older. Overall, higher scores reflect greater fear of hypoglycaemia, a higher score on the Behaviour Subscale reflects a greater tendency to avoid hypoglycaemia and/or its negative consequences, and a higher score on the Worry Subscale indicates more worry concerning episodes of hypoglycaemia and its consequences. The CHFS has shown adequate internal consistency (HFSC behaviour subscale alpha = 0.70; CHFS worry subscale alpha = 0.89; and CHFS-Total alpha = 0.85) [38]. HFSC worry subscale and total scores have been shown to correlate significantly with other measures of anxiety [38]. Total scores and subscale scores will be calculated as z-scores standardised to the instrument-specific and baseline means and standard deviations.

The Self-Efficacy for Diabetes Self-Management (SEDM) is a 10-item self-report questionnaire for youth aged 10–16 years that examines confidence to carry out self-care behaviours and covers all the key areas of diabetes self-management [39]. The SEDM will be completed by participants who are 10 years and older. Participants are asked “How sure are you that you can do each of the following, almost all the time” and items are rated from 1 (not at all sure) to 10 (completely sure) and averaged. Higher scores indicate higher self-efficacy. The SEDM has demonstrated good validity and reliability (Cronbach’s alpha 0.9) [39].

At Visit 1, parents/guardians of enrolled participants who provide written consent for their own participation in the study will complete a short questionnaire collecting demographic characteristics (e.g., age, gender, education level, and ethnicity). At the baseline and follow-up visits parents/guardians will complete questionnaires to assess their perceptions of their own fear of their child experiencing hypoglycaemia using the parent version of the scale [38].

### Statistical analysis

A sample size of 88 (44 participants in each group) would provide 80% power to detect a difference in changes in HbA1c of 7 mmol/mol (0.75%) between the intervention and control group using standard deviation of 15 mmol/mol and correlation of 0.7 between repeated observations on the same person and a two-sided test at the 0.05 level [26, 40]. This is a clinically important difference and similar to other proven technologies such as insulin pumps or CGM. To account for a small amount of missing data and loss to follow-up, we will recruit a sample size of 100 (50 participants per group) at baseline.

The statistician will be blinded to allocation arm and will use non-informative group codes until all planned analyses are completed. Descriptive statistics will be calculated for all variables. The primary analysis will follow the intention-to-treat principle with all participants analysed in the group to which they were randomised, regardless of actual sensor wear. Additional analyses include: HbA1c, glucose monitoring frequency and adherence, episodes of moderate and severe hypoglycaemia (as defined in Safety section below), episodes of DKA, and psychosocial variables using Poisson and linear mixed models as appropriate. Statistical analysis will be performed using Stata software with two-sided p < 0.05 considered significant.

### Safety

For safety monitoring purposes, LibreView reports will be produced at 2-, 4-, 8-, and 12-weeks from isCGM commencement and checked for episodes of moderate (blood glucose values ≤ 3.9 mmol/L) and severe (child is having altered mental status and unable to assist in their care or is semiconscious or unconscious) hypoglycaemia. In the event the proportion of ‘low’ values is > 4% or ‘very low’ values is > 1% the report will be forwarded to the participant’s usual diabetes care provider for follow-up. Sensor failure rates and cutaneous adverse events (e.g., pain, itching, redness, subcutaneous haemorrhage, infection) will be self-reported to research staff at each visit or by phone call every four weeks throughout the study. All adverse events will be recorded in an Adverse Event form.

Participants will be asked to contact research staff immediately (by sending a photo of their affected skin site, if possible) if they notice a cutaneous issue associated with wearing the sensor. Clinical research staff will then advise if medical treatment is necessary. Participants will be referred to their general practitioner or emergency department, as appropriate, for management of medical events.

For more significant or persistent adverse events involving skin, a barrier product will be offered (e.g., Cavilon spray, SkinTac™) or drug therapy (e.g., zinc ointment, Fenistil gel, or hydrocortisone cream) prescribed, and the participant’s caregiver will be instructed to relocate the sensor to another area of the skin such that the effects are maintained at a tolerable level. Ultimately, the decision to continue or discontinue the use of the FreeStyle Libre 2 when localised skin symptoms occur will be made in consultation with the participant.

An internal Safety Monitoring Committee will be notified of severe adverse events (e.g., severe hypoglycaemia [BG value ≤ 3.9 mmol/L and resulting in loss of consciousness, a call for an ambulance and/or admission to hospital, or use of glucagon], DKA [being unwell due to hyperglycaemia and high ketones, and requiring a visit to the doctor, emergency room, or admission to hospital]) immediately after being reported to research staff. The Committee will then discuss any necessary action. Non-urgent events (moderate events) will be reported to the lead investigator after being reported to research staff. The internal Safety Monitoring Committee will be comprised of clinical investigators (CJ, BW, EW, AL, VC).

### Data management

All study participants will be assigned a non-informative study identification number. Only research staff and investigators will have access to the electronic study records for the purposes of recording data and checking completeness of data. Data will be recorded and stored electronically in REDCap, which is securely hosted at the University of Otago. Identifiable information (e.g., date of diagnosis, address, date of birth) will not be stored in REDCap. Instead, age in whole numbers and duration of diabetes in whole numbers will be recorded in REDCap. Local sites will, however, hold in locked Excel sheets their own participants with address and contact details (phone number and emails), so that if the local sites need to contact participants (for replacement Libre 2 devices etc.) they can do so.

REDCap features (e.g., calendar and colour-coding forms to indicate complete or missing data) will help ensure adherence to timeframes, compliance to measurement procedures, and completeness of data. Data will be routinely checked for missing and/or erroneous values by the study coordinator. At the end of the study, original data collection sheets and written informed consent will be stored securely at the lead site along with copies of all data collected electronically. The lead investigator will retain an anonymised electronic copy of the cleaned data set, with all identifying information removed. The data set may be shared as part of the scientific peer-review process or shared to conduct a meta-analysis (e.g., impacts of flash glucose monitoring on glycaemic control). The electronic dataset will be destroyed 10 years from the end of the study.

## Discussion

isCGM technology has the potential to significantly improve diabetes control in children, and limited data is available especially for the second-generation isCGM system. Increasing time in range, reducing HbA1c, reducing burden, and improving quality of life for children with this lifelong chronic disease is important and improving glycaemic control reduces the risk of acute and chronic diabetes complications. If next generation isCGM is effective in an RCT, it will then increase our ability to have this device available and funded for children worldwide.

## Data Availability

De-identified data related to the primary and secondary outcomes will be available to those involved in the peer review process for publication in a scientific journal, upon request.

## Abbreviations

BG: Blood glucose
BMI: Body mass index
CGM: Continuous glucose monitoring
DHB: District health board
DKA: Diabetic ketoacidosis
HbA1c: Glycated haemoglobin
isCGM: Intermittently scanned continuous glucose monitoring
RCT: Randomised controlled trial
SMBG: Self-monitoring blood glucose
